# Prevalence and factors associated with visual impairment in middle-aged and older Chinese population

**DOI:** 10.3389/fmed.2022.962729

**Published:** 2022-11-28

**Authors:** Hanyuan Ye, Yun Zeng, Hongxia Xiao, Jing Yu, Yun Liu, Shuang Zhang, Bingjie Zhang

**Affiliations:** Department of Ophthalmology, Jingmen No.2 People's Hospital, Jingmen, China

**Keywords:** visual impairment (VI), prevalence, risk factors, cross-sectional studies (MeSH), China

## Abstract

**Objective:**

This study aimed to estimate the prevalence of visual impairment and to identify the factors associated with it in China.

**Methods:**

Data for this cross-sectional study were retrieved from the China Health and Retirement Longitudinal Study (CHARLS) for a total of 16,480 subjects who completed the questionnaire between June 2011 and March 2012. The prevalence of visual impairment was estimated considering the complex survey design and response rate. Associated factors were identified using the weighted logistic regression analysis.

**Results:**

The overall prevalence of visual impairment among middle-aged and older Chinese adults was 6.22%. Regionally, Qinghai and Gansu provinces showed the highest prevalence of visual impairment, whereas Shanghai showed the lowest prevalence of visual impairment in China. Older age (OR = 1.888; 95% CI: 1.537–2.467) was correlated with a higher likelihood of visual impairment, whereas a non-alcohol intake (OR = 0.072; 95% CI: 0.018–0.246) was correlated with a lower likelihood of visual impairment. Hypertension (OR = 1.299; 95% CI: 1.189–1.467), diabetes (OR = 2.000; 95% CI: 1.163–3.765), lung diseases (OR = 1.795; 95% CI: 1.067–3.019), liver diseases (OR = 1.270; 95% CI: 1.221–2.876), stroke (OR = 1.135; 95% CI: 1.107–3.528), and heart disease (OR = 1.350; 95% CI: 1.104–1.708) were significantly associated with visual impairment.

**Conclusion:**

Geographical variations in the prevalence of visual impairment in China were defined, indicating that such variations do exist in China. Age, alcohol intake, hypertension, diabetes, lung diseases, liver diseases, stroke, and heart disease were factors associated with visual impairment.

## Introduction

Visual impairment is an important global public health issue. It leads to impaired socioeconomic status, reduced quality of life, and an increased risk of indirect hospitalization and death (1, 2). According to the Global Burden of Diseases, Injuries, and Risk Factors Study 2019, ~328.77 million people worldwide are affected by blindness or severe or moderate visual impairment (3). As the most populous country in the world, China has the highest population with visual impairment, which imposes an enormous economic burden on such individuals and their families and the public healthcare system (4). Given the morbidity, mortality, and socioeconomic burden associated with visual impairment, it is vital to understand the risk factors for visual impairment so that targeted preventive measures can be employed.

The incidence of visual impairment is strongly correlated with increased age, especially after 50 years (1). One study reported that 6.05–15.3% of people in rural areas have a visual impairment in China (5). With the aging of the population, the prevalence of visual impairment continues to grow. It has been predicted that China will account for more than 25% of the world's aging population by 2040, by which point the number of people with visual impairment will reach more than 100 million (6, 7). According to data from the World Health Organization, the proportion of older people aged ≥60 years exceeds 30% in Japan (8). Notwithstanding, Korea is one of the countries with the highest growth rate of the elderly population. According to statistics published by the Korea National Statistical Office, there is a steady growth in the proportion of older adults aged ≥65 years in Korea, and this growth is expected to increase to 24.3% by 2030, at which time Korea will become a “super-aged society” (9). Countries in the East Asia region are thus also facing increased pressure from the visually impaired population.

Previous studies have identified that advanced age, female sex, low socioeconomic status, low education level, memory decline, depression, temporary housing, obesity, and having chronic diseases, such as hypertension or diabetes, as risk factors are associated with visual impairment (10–12). Most such studies were conducted on relatively small regional populations (13, 14), yet the prevalence of visual impairment varies markedly between populations (13, 15–17). The epidemiological and geographical variations of visual impairment among the older population in China remain unclear. A comprehensive overview with an updated epidemiological study is essential for policymakers to develop strategies to better allocate resources and relieve the burden on the public healthcare system. This study aimed to determine the prevalence and factors associated with visual impairment in a large, population-based sample derived from the China Health and Retirement Longitudinal Study (CHARLS).

## Methods

### Study population

The China Health and Retirement Longitudinal Study is a nationally representative longitudinal survey of people aged 45 years and older in China (18). It was conducted by the China Center for Economic Research at Peking University to investigate the prevalence, trajectories, and associated risk factors of various age-related phenotypes in the Chinese middle-aged and elderly population. Details of the sampling procedure have been published elsewhere (18, 19). In brief, the CHARLS baseline survey covered 450 villages and settlements in 150 counties and districts in China. All the participants were sampled in four stages, namely county, village, family, and individual sampling. In total, 17,705 individuals from 10,257 families were interviewed, and the survey sample generally represented the middle-aged and elderly population in China. Trained interviewers conducted face-to-face and computer-assisted family interviews using structured questionnaires. The CHARLS project comprised a national baseline survey from 2011 to 2012 and was followed up every 2 years. Anthropometric and other physical measurements, including height, weight, and blood pressure, were measured by the interviewers. In this study, data from the 2011 CHARLS baseline survey were collected.

This study was approved by the Institutional Review Board and Ethics Committee of Peking University. Informed consent was obtained from all participants, adhering to the tenets of the Declaration of Helsinki.

### Visual impairment assessment

The visual impairment conditions of the respondents were assessed based on their self-reported responses to the following question: “Do you have a vision problem?” The respondents were classified as having visual impairment if they responded “Yes” to this question.

### Control variables

The prevalence of visual impairment in various geographical locations was categorized by province. Ningxia, Tibet, Hainan, Taiwan, Hong Kong, and Macau were not included in this survey. Information on age, gender, education level (primary or below, middle school, high school, or college/above), marital status (married/partnered or otherwise), smoking status (yes or no), and drinking (drinking alcohol more than once a month, less than once a month, or not at all) were collected by trained interviewers who used the 2011 CHARLS questionnaire. Residential areas were considered either urban or rural based on the criteria designed by the National Bureau of Statistics. Health status information pertaining to cancer or malignant tumor, lung disease, liver disease, stroke, and heart disease was collected *via* self-reports. Hypertension was defined as blood pressure ≥140/90 mmHg at baseline or self-reported use of antihypertensive medication. Diabetes was defined as a self-reported diagnosis of diabetes or fasting plasma glucose of ≥7.0 mmol/L or glycated hemoglobin levels of ≥6.5%. Dyslipidemia was identified as total cholesterol (TC) of ≥6.22 mmol/L (240 mg/dL), triglycerides (TG) of ≥2.26 mmol/L (200 mg/dL), high-density lipoprotein cholesterol (HDLC) of <1.04 mmol/L (40 mg/dL), low-density lipoprotein cholesterol (LDLC) of ≥4.14 mmol/L (160 mg/dL), or self-reported dyslipidemia in accordance with the Chinese guidelines on the prevention and treatment of dyslipidemia in adults.

### Serum measurements

Trained nurses at township hospitals or the local offices of the Chinese Center for Disease Control (CDC) collected 8 mL of fasting blood specimens from each participant after they had fasted overnight for at least 8 h. The whole blood and centrifuged serum were transported to the Chinese CDC at −20°C and stored at −80°C. Afterward, the plasma glucose and lipids were measured at the Capital Medical University laboratory.

### Statistical analysis

The prevalence of visual impairment was calculated with due consideration of the complex survey design and non-response rate. The overall prevalence of visual impairment was calculated by applying the inverse probability weighting method using the Proc Surveyfreq procedure. The χ^2^ test was used to compare the differences between the categorical variables. Two models were built using the Proc Surveylogistic procedure to explore the factors associated with visual impairment. The variables in Model 1 included age, sex, residence, education, marital status, drinking, smoking status, and medical insurance. The variables in Model 2 included the variables in Model 1 as well as a history of chronic diseases, such as hypertension, diabetes, dyslipidemia, cancer or malignant tumor, lung disease, liver disease, stroke, and heart disease. The odds ratios (ORs) and 95% confidence intervals (CIs) were demonstrated for the variables in the models. All the analyses were conducted using SAS 9.4 for Windows (SAS Institute Inc., Cary, North Carolina, USA), and a *p*-value (two-tailed) of < 0.05 was considered statistically significant.

## Results

### Group comparisons of sociodemographic and clinical variables

After filtering, a total of 16,480 subjects were deemed eligible for this study. Of these, 1,025 subjects (47.8% women and 52.1% men) had visual impairment. Table 1 shows comparisons of the sociodemographic and clinical variables between the subjects with and without visual impairment, as well as the significant differences in age, medical insurance, hypertension, diabetes, lung disease, liver disease, stroke, and heart disease (all *p-*values of <0.05). All these significant variables were included in the subsequent analysis as potential risk factors.

**Table 1:** Characteristics of the respondents by visual impairment in cross-sectional study (values are the weighted percentage of vision problem).

**Variables**	**Vision problem**		***P*-value**
	**Yes (*n* = 1,025)**	**No (*n* = 15,455)**	
Gender			0.368
Male	47.8%	47.9%	
Female	52.1%	52.1%	
Age, years			< 0.01
45–59	57.2%	72.2%	
≥60	42.8%	27.8%	
Residence			0.543
Rural	83.6%	81.0%	
Urban	16.4%	19.0%	
Education			0.961
Primary or below	67.3%	66.4%	
Middle school	20.3%	20.8%	
High school	7.6%	7.9%	
College or above	4.8%	4.9%	
Marital status			0.533
Married or partnered	86.5%	87.2%	
Otherwise	13.5%	12.8%	
Smoking			0.373
Yes	40.6%	39.2%	
No	59.4%	60.8%	
Drinking			0.085
Drink more than once a month	23.5%	25.1%	
Drink but less than once a month	6.5%	8.0%	
None	69.9%	66.9%	
Medical insurance			< 0.01
Yes	94.6%	93.2%	
No	5.4%	6.8%	
Hypertension			< 0.01
Yes	31.2%	24.0%	
No	68.8%	76.0%	
Diabetes			< 0.01
Yes	9.5%	5.5%	
No	90.5%	94.5%	
Dyslipidemia			0.339
Yes	10.2%	9.3%	
No	89.8%	90.7%	
Cancer or malignant tumor			0.148
Yes	1.5%	1.0%	
No	98.5%	99.0%	
Lung disease			< 0.01
Yes	17.4%	9.6%	
No	82.6%	90.4%	
Liver disease			0.02
Yes	5.2%	3.7%	
No	94.8%	96.3%	
Stroke			< 0.01
Yes	5.1%	2.2%	
No	94.9%	97.8%	
Heart disease			< 0.01
Yes	17.1%	11.5%	
No	82.9%	88.5%	

### Prevalence of visual impairment

**Figure 1:**
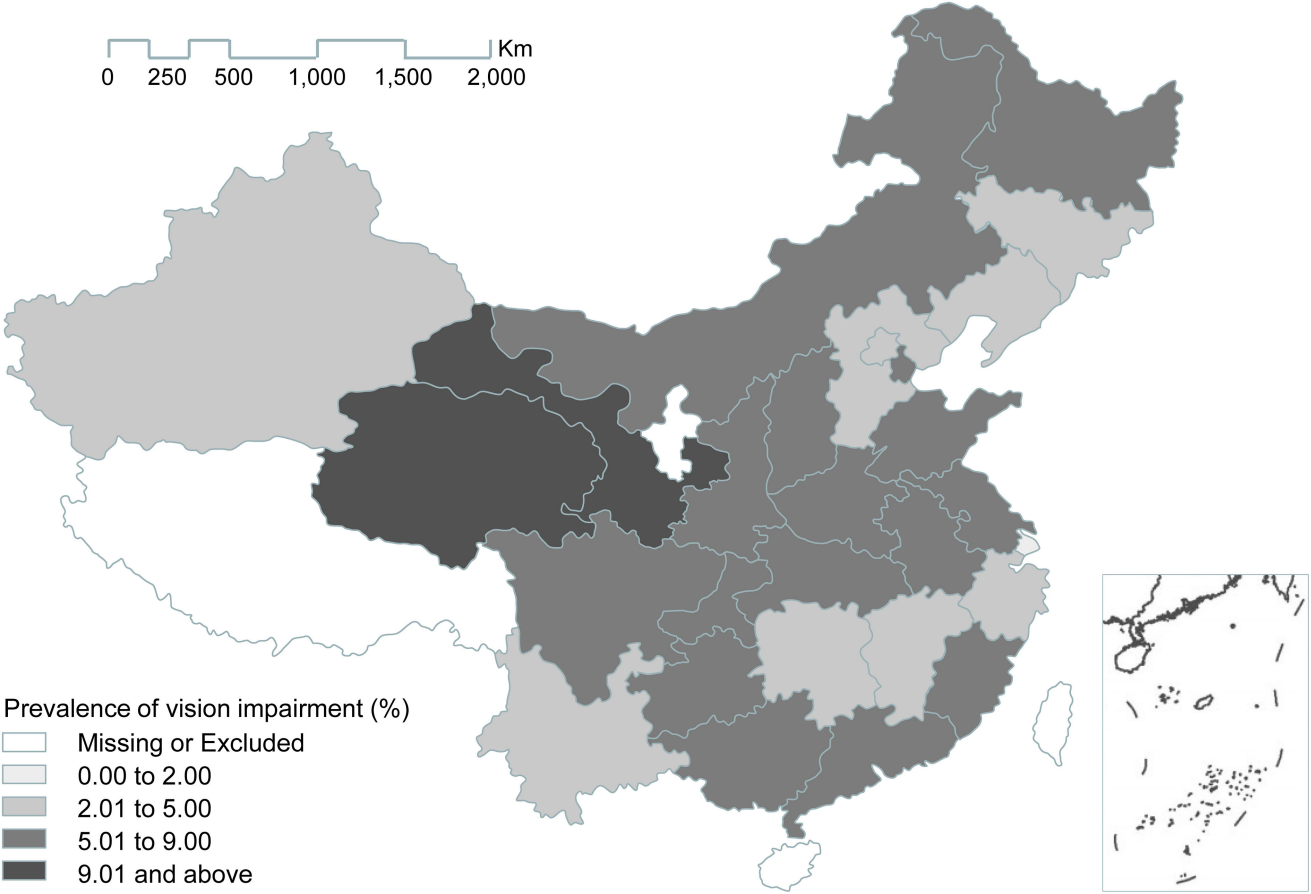
Prevalence of visual impairment in different provinces of China.

Among all the included respondents, 1,025 had visual impairment. The overall and sex-specific prevalence of visual impairment among the respondents are displayed in Table 2. The overall prevalence of visual impairment was 6.22% (6.21% women and 6.23% men). As expected, the incidence rate for the subjects aged 60 years and older was higher, at up to 8.50%, while the incidence rate for the subjects aged 45–59 years was only 5.1%. The prevalence of visual impairment was similar for the different residences, education levels, marital statuses, and smoking statuses of the subjects. The prevalence of visual impairment was much higher in those with chronic diseases (i.e., hypertension, diabetes, cancer or malignant tumor, chronic lung disease, stroke, and/or heart disease) than in those without these systemic diseases, and this trend was similar for both men and women. The prevalence of visual impairment in each province was calculated and visualized on a map (Figure 1). The map showed that one province, Shanghai, had the lowest prevalence (<2.00%), while the two provinces with the highest prevalence were Qinghai and Gansu (>9.01%).

**Table 2:** Prevalence of self-reported history of visual impairment by age, residence, education, marital status, smoking, drinking, chronic diseases, and medical insurance (values are the weighted prevalence of visual impairment).

**Variables**	**Prevalence**		
	**Women**	**Men**	**Total**
Total	6.21%	6.23%	6.22%
Age, years
45–59	5.3%	5.0%	5.1%
≥60	8.0%	9.1%	8.5%
Residence
Rural	5.2%	6.4%	5.7%
Urban	5.5%	3.9%	4.8%
Education
Primary or below	6.5%	5.9%	6.3%
Middle school	5.4%	6.5%	6.1%
High school	4.6%	7.0%	6.0%
College or above	5.7%	6.2%	6.0%
Marital status
Married or partnered	6.3%	6.1%	6.2%
Otherwise	6.0%	7.5%	6.5%
Smoking
Yes	6.6%	6.3%	6.4%
No	6.0%	6.2%	6.1%
Drinking
Drink more than once a month	5.9%	5.8%	5.9%
Drink but less than once a month	5.2%	5.1%	5.2%
None	6.5%	6.5%	6.5%
Medical insurance
Yes	6.3%	6.3%	6.3%
No	5.4%	4.5%	5.0%
Hypertension
Yes	7.7%	8.2%	7.9%
No	5.7%	5.5%	5.6%
Diabetes
Yes	10.9%	9.5%	10.2%
No	6.0%	6.0%	6.0%
Dyslipidemia
Yes	7.0%	6.4%	6.7%
No	6.1%	6.1%	6.1%
Cancer or malignant tumor
Yes	8.5%	9.3%	8.9%
No	6.2%	6.2%	6.2%
Lung disease
Yes	10.0%	11.4%	10.7%
No	5.8%	5.6%	5.7%
Liver disease
Yes	7.6%	9.4%	8.5%
No	6.2%	6.1%	6.1%
Stroke
Yes	11.6%	14.8%	13.3%
No	6.1%	6.0%	6.0%
Heart disease
Yes	9.6%	8.3%	8.9%
No	5.8%	5.9%	5.9%

### Factors associated with visual impairment

In the univariate weighted analyses, age, medical insurance, hypertension, diabetes, lung disease, liver disease, stroke, and heart disease were significantly associated with visual impairment (Table 1). In the multivariate weighted analyses, after adjusting for other confounding factors, age, drinking, medical insurance, hypertension, diabetes, lung disease, liver disease, stroke, and heart disease remained significantly associated with visual impairment (Table 3). Subjects aged 60 years and older were associated with a higher visual impairment risk of 1.888-fold (95% CI: 1.537–2.467) than the subjects aged between 45 and 59 years. Compared with those who drank alcohol more than once a month, the adjusted OR of those who did not drink alcohol at all was 0.195 (95% CI: 0.069–0.545) in Model 1 and 0.072 (95% CI: 0.018–0.246) in Model 2. Compared to those with medical insurance, the subjects without medical insurance had a higher risk of glaucoma, and the adjusted OR was 4.565 (95% CI: 1.114–18.715). The subjects with chronic diseases, such as hypertension (OR = 1.299; 95% CI: 1.189–1.467), diabetes (OR = 2.000; 95% CI: 1.163–3.765), lung disease (OR = 1.795; 95% CI: 1.067–3.019), liver disease (OR = 1.270; 95% CI: 1.221–2.876), stroke (OR = 1.135; 95% CI: 1.107–3.528), or heart disease (OR = 1.350; 95% CI: 1.104–1.708), had a higher risk of glaucoma.

**Table 3:** Cross-sectional analysis of visual impairment with different variables in different models.

**Variables**	**Cross-sectional analysis results**			
	**Model 1**		**Model 2**	
	**OR**	**95%CI**	**OR**	**95%CI**
Gender
Male				
Female	1.174	0.781–1.764	1.118	0.739–1.691
Age, years
45–59				
≥60	1.922	1.561–2.516	1.888	1.537–2.467
Residence
Rural				
Urban	1.144	0.672–1.946	1.102	0.642–1.891
Education
Primary or below				
Middle school	0.938	0.498–1.768	0.883	0.457–1.704
High school	0.946	0.477–1.876	0.943	0.464–1.919
College or above	1.248	0.509–3.059	1.196	0.481–2.974
Marital Status
Married or partnered				
Otherwise	0.896	0.511–1.571	0.882	0.502–1.549
Smoking
Yes				
No	1.316	0.868–1.996	1.233	0.806–1.886
Drinking
Drink more than once a month				
Drink but less than once a month	0.360	0.140–2.202	0.288	0.190–2.099
None	0.195	0.069–0.545	0.072	0.018–0.246
Medical insurance
Yes				
No	4.617	1.128–18.895	4.565	1.114–18.715
Chronic diseases
Hypertension (yes vs. no)			1.299	1.189–1.467
Diabetes (yes vs. no)			2.000	1.163–3.765
Dyslipidemia (yes vs. no)			1.079	0.573–2.030
Cancer or malignant tumor (yes vs. no)			0.672	0.088–5.117
Lung disease (yes vs. no)			1.795	1.067–3.019
Liver disease (yes vs. no)			1.270	1.221–2.876
Stroke (yes vs. no)			1.135	1.107–3.528
Heart disease (yes vs. no)			1.350	1.104–1.708

Model 1: Adjusted for age, sex, residence, education, marital status, drinking, smoking, and medical insurance.

## Discussions

Due to the growth and aging of the global population, many aged individuals experience visual impairment, which presents a huge challenge to public health systems worldwide. This study aimed to elucidate the prevalence and distribution of visual impairment and its potential risk factors in China using a nationally representative longitudinal survey. This study found that a population-adjusted prevalence of visual impairment among adults aged 45–59 years was 5.1%, while for those aged 60 years and older, it was 8.5%. There was an increasing trend in the prevalence of visual impairment with advancing age. We further found a higher prevalence trend of visual impairment among patients with chronic diseases, such as hypertension, diabetes, lung disease, stroke, and heart disease, and patients with diabetes had an almost double risk of visual impairment.

The prevalence of visual impairment varies according to different surveys in different countries. In the United States, estimates of the prevalence of visual impairment have been shown to vary between 1.6 and 24.8% for those younger than 65 years and between 2.2 and 26.6% for those 65 years or older (20). In Australia, the reported incidence of unilateral visual impairment was 14.6% among non-indigenous Australians (21). Another study from Asia, namely, the Singapore Indian Eye Study, showed that 4.8% of the population aged 40 years and older had visual impairment (22). The overall prevalence of visual impairment in this study, therefore, appears different than that in other studies, although direct comparisons are not possible due to differences in age compositions, study designs, and ethnic, health, and economic conditions.

We further observed geographical variations in the prevalence of visual impairment. Our results showed that Shanghai had the lowest prevalence of visual impairment (<2.00%), while two provinces, Qinghai and Gansu, displayed the highest prevalence. Shanghai, as the most developed region in China, has the lowest incidence of visual impairment, most likely due to the availability of good medical treatments and its residents' high levels of education, which likely contribute to preventing or delaying visual impairment. Qinghai and Gansu provinces, which are located in Western China, have the highest prevalence of visual impairment, most likely due to a shortage of healthcare services as the populations of less developed and more geographically remote places lack health awareness, which is an important factor among Western China residents. In addition, individuals living in Qinghai may experience higher ambient ultraviolet radiation as the region is located on the world's highest plateau, and residents are therefore particularly prone to cataracts.

In this study, as expected, similar to most other studies, age was a risk factor for visual impairment (4, 21). It is easy to understand that as people age, the probability of suffering from age-related ocular diseases that cause visual impairment, such as cataracts, glaucoma, and age-related macular degeneration, increases significantly. However, in this study, gender was not associated with the incidence of visual impairment, which is consistent with the study by Marmamula (23), but in contrast to that reported by Bourne et al. (24). Our results also showed that individuals with some chronic diseases, especially diabetes, hypertension, and stroke, were more likely to have a visual impairment. Our findings further support those of other studies that have shown equivalent results with respect to different regions and ethnic groups (25, 26). For example, the population-based data from the National Health and Nutrition Examination Survey showed that individuals with diabetes have higher rates of visual impairment than those without (25). It is no coincidence that our finding is also consistent with Yonekawa's report that diabetes is independently associated with visual impairment (27). The reason for visual impairment in diabetes is that individuals with diabetes are more prone to developing multiple ophthalmic conditions, including diabetic retinopathy (28), cataract (29), glaucoma (30), macular edema (31), and retinal vein occlusion (32). In terms of systemic hypertension and stroke, which have also been identified as contributory risk factors for visual impairment, numerous studies have demonstrated that systemic hypertension likely leads to hypertensive retinopathy, fundus hemorrhage, and retinal vein occlusion, which consistently affect visual function (33, 34). It has similarly been reported that patients with stroke have a higher prevalence of glaucoma and retinal vascular occlusion (35–37). Furthermore, up to 8–25% of patients who have had a stroke may develop visual field loss because of the involvement of the visual pathway or visual cortex (38).

The strength of this study lies in the acquisition of data from a nationwide, population-based survey. In addition, the application of multivariate analysis in our research may be the reason our results differ partially from those of previous studies; the application of multivariate analysis in two models provided a reliable assessment of the relationship between the predicted risk factors and visual impairment. In addition, global positioning system matching, data checking, recording, interview checking, and calling participants back were implemented at every stage of the study to ensure data quality and reliability.

However, several limitations should be considered. First, although the method has been widely used in many population-based studies, all the information for visual impairment was based on self-reports, which are less accurate and reliable than more objective measurements of visual impairment and may lead to potential bias (39, 40). As a result, the prevalence may not be representative of the general population. Future studies may consider using more objective indicators to measure visual impairment. Second, we did not distinguish between different visual diseases for visual impairment, which may have affected the prevalence in a variety of ways. Third, although we tried our best to control for as many covariates as possible, such as age, sex, residence, education level, marital status, and some chronic diseases, other covariates not included in our study may affect visual impairment. Finally, since the survey was conducted in China, the findings of this study are of significance only to the Asian population.

## Conclusion

In this study, we described the prevalence and geographical variations of visual impairment in China using data from a nationally representative longitudinal survey. Our study indicated that the prevalence of visual impairment remains high, with variable prevalence in different provinces. This information may assist policymakers in provinces with high incidences of visual impairment to focus on this condition and thus help control it better. The risk factor analysis demonstrated that drinking alcohol and having certain chronic diseases rendered an individual more likely to suffer from the onset of visual impairment, which suggests that changes in lifestyle and behaviors and the aggressive prevention, diagnosis, and treatment of systemic diseases are likely to reduce the burden of visual impairment. Additional studies with comprehensive ophthalmic examinations are needed to adequately characterize visual impairment and better understand the factors influencing it.

## Data availability statement

Publicly available datasets were analyzed in this study. This data can be found here: http://charls.pku.edu.cn/.

## Ethics statement

The studies involving human participants were reviewed and approved by Institutional Review Board and Ethics Committee of Peking University. The patients/participants provided their written informed consent to participate in this study.

## Author contributions

HY and BZ designed the research. YZ, HX, JY, YL, and SZ analyzed the data. HY drafted the manuscript. All authors read and approved the final manuscript.

## Conflict of interest

The authors declare that the research was conducted in the absence of any commercial or financial relationships that could be construed as a potential conflict of interest.

## Publisher's note

All claims expressed in this article are solely those of the authors and do not necessarily represent those of their affiliated organizations, or those of the publisher, the editors and the reviewers. Any product that may be evaluated in this article, or claim that may be made by its manufacturer, is not guaranteed or endorsed by the publisher.
